# Contribution of trastuzumab to the prognostic improvement of HER2-positive early breast cancer in Spain: an estimation of life years and disease-free life years gained since its approval

**DOI:** 10.18632/oncotarget.27039

**Published:** 2019-07-02

**Authors:** Eva Ciruelos, Emilio Alba, Rafael López, Anna Lluch, Miguel Martín, Isabel Arroyo, Beatriz Navarro, David Carcedo, Ramón Colomer, Joan Albanell

**Affiliations:** ^1^ Department of Medical Oncology, Hospital Universitario 12 de Octubre, Madrid, Spain; ^2^ Clinical Oncology Unit, Hospitales Universitarios Regional y Virgen de la Victoria; Instituto de Investigación Biomédica de Málaga (IBIMA), Malaga, Spain; ^3^ Department of Medical Oncology, Hospital Clínico Universitario e Instituto de Investigación Sanitaria-CIBERONC, Santiago de Compostela, Spain; ^4^ Department of Medical Oncology, Hospital Clínico Universitario de Valencia, Health Research Institute INCLIVA, University of Valencia, The Centre of Networked Biomedical Cancer Research (CIBERONC), Valencia, Spain; ^5^ Medical Oncology Service, Instituto de Investigación Sanitaria Gregorio Marañón, Universidad Complutense, GEICAM, CIBERONC, Madrid, Spain; ^6^ Department of Payer Evidence and Health Economics, Roche Pharma, Madrid, Spain; ^7^ Medical Department, Roche Pharma, Madrid, Spain; ^8^ Oblikue Consulting, Barcelona, Spain; ^9^ Medical Oncology Service, Hospital Universitario La Princesa, Madrid, Spain; ^10^ Medical Oncology Service, Hospital del Mar, IMIM, Universitat Pompeu Fabra, The Centre of Networked Biomedical Cancer Research (CIBERONC), and Centro Oncológico Clara Campal-HM Delfos, Barcelona, Spain

**Keywords:** early breast cancer, trastuzumab, life-years, economics, Spain

## Abstract

Introduction: Trastuzumab has become the standard treatment for both HER2-positive early and metastatic breast cancer (HER2+ eBC or mBC) since its approval. The objective of the study is to estimate the benefit of adjuvant trastuzumab in the treatment of patients with HER2+ eBC in terms of life years gained (LYG) and disease-free life years gained (DFLYG) since its approval in Spain in 2006.

Results: 35,851 women make up the cohorts from 2006 to 2017. In the T (trastuzumab)+CT (chemotherapy) scenario, the sum of life years was 605,358 (525,964 disease-free) versus 564,137 (489,916 disease-free) in the CT scenario, resulting in 41,221 LYG (36,048 disease-free) due to trastuzumab. The general population for the same age range would have generated 704,331 LY. The estimated incremental cost was 880.43 million€ (€24,558.13 per patient) from 2006 to 2035. The incremental cost-effectiveness ratios obtained were €20,644 and €23,960 per LYG and DFLYG, respectively.

Methods: An epidemiological model was developed with a time horizon until 2035 and a 3% discount rate. The model compared two scenarios, with and without trastuzumab as adjuvant therapy. The effectiveness data to model the survival curves were obtained from BCIRG 006 study and direct costs were included.

Conclusions: Adjuvant trastuzumab has substantially improved the survival of patients with HER2+ eBC, contributing over 41,000 LYG to Spanish society (over 36,000 DFLYG) in a cost-effective manner. However, the sum of LYG with trastuzumab is still far from the LY estimated for the general population, supporting the need of further advances in HER2+ eBC.

## INTRODUCTION

Breast cancer (BC) is considered a public health problem due to its high incidence, prevalence and mortality [1]. In fact, BC is the most frequent and fatal malignancy in Spanish women, with 27,747 cases diagnosed in 2015, and an age-adjusted mortality rate of 15.9/100,000 [2].

Breast cancer is a very heterogeneous disease with a number of subtypes, based on the over-expression of hormone receptors or the overexpression or amplification of human epidermal growth factor receptor 2 (HER2). Both factors, hormone receptors and HER2, dictate the systemic treatment options of the patients. The prognosis and treatment of the disease depend on the stage (TNM classification: *Tumor–Node–Metastases*), site of metastasis and patient co-morbidities.

Historically, breast cancer patients with HER2 positive tumours, which account for approximately 15–20% of breast cancers, had a poorer prognosis than HER2 negative tumours. However, in women with HER2+ BC, the approval of the anti-HER2 monoclonal antibody trastuzumab in the year 2000 for the treatment of metastatic BC and in 2006 for the treatment of early BC considerably improved the prognosis of the disease - the drug becoming the standard of treatment for HER2+ BC in both the metastatic and early disease settings. The management of early BC is more homogeneous than that of metastatic BC, the latter being influenced by the previous treatment received, the disease burden, concomitant diseases, the site of metastasis, and the molecular phenotype of the metastatic tumour cells [3]. For this reason, and because it is a curative setting, the present analysis focuses on early stage disease.

Although there is ample evidence of the clinical benefits of trastuzumab as adjuvant therapy in HER2+ eBC [4–10], no studies have estimated the added value of the treatment in the real life clinical practice setting in Spain.

Establishing the contribution of therapeutic procedures in real life clinical practice is one of the main challenges for both the healthcare system [11] and healthcare professionals [12].

The primary objective of the present study was to estimate the benefits of trastuzumab in the treatment of women with HER2+ eBC in terms of life years gained (LYG) and disease-free life years gained (DFLYG), since approval of the drug in Spain in 2006. As secondary objective, we aimed to estimate the additional cost for the healthcare system of treatment with trastuzumab, and to compare both parameters in added terms for the Spanish population during that period of time.

## RESULTS

### Epidemiological results

The incidence outcomes of the epidemiological model show an increase in the diagnosed cases of BC, from 21,132 new diagnoses in 2006 to 24,994 in 2017. This estimate is consistent with the data reported by GLOBOCAN [13], and slightly below the estimate of the Spanish Cancer Registries Network (REDECAN) (25,215 new cases in 2015) [14]. Table 1 shows the evolution from 2006 to 2017 of the new cases of BC in general and the women with HER2+ eBC selected for analysis. The observed increase in diagnosed cases is attributable to variations in the gender and age pyramid of the Spanish population, which clearly aged over the entire period.

**Table 1:** Estimation of the cohorts of women from 2006 to 2017 included in the model

Costs	2006	2007	2008	2009	2010	2011	2012	2013	2014	2015	2016	2017
Total BC(25–90 years)	21,132	21,608	22,101	22,533	22,925	23,291	23,651	23,936	24,175	24,442	24,724	24,994
Target population^*^	**2,664**	**2,724**	**2,787**	**2,841**	**2,891**	**2,937**	**2,982**	**3,018**	**3,249**	**3,377**	**3,173**	**3,208**

^*^After selecting women with HER2+ early breast cancer; BC: breast cancer.

### Primary objective: clinical results

The sum of life years (LY) of the women with HER2+ early BC during the analytical period for all the cohorts was 605,358 years in the trastuzumab + chemotherapy (T+CT) scenario, versus 564,137 in the hypothetical scenario of the absence of trastuzumab as adjuvant therapy (CT) since 2006. Therefore, the addition of trastuzumab to CT was estimated to afford a total of 41,221 LYG for the Spanish society.

In order to place these results in context, the general population without HER2+ eBC for the same age range during the analytical period would have generated 704,331 life years. This implies a difference of 98,973 life years between the general population without disease and the T+CT scenario, and of 140,194 life years between the general population and the CT scenario.

With regard to the sum of disease-free life years (DFLY), the total DFLY was 525,964 in the T+CT scenario, versus 489,916 in the CT scenario. Thus, the addition of trastuzumab to CT would afford a total of 36,048 additional disease-free life years.

**Figure 1:**
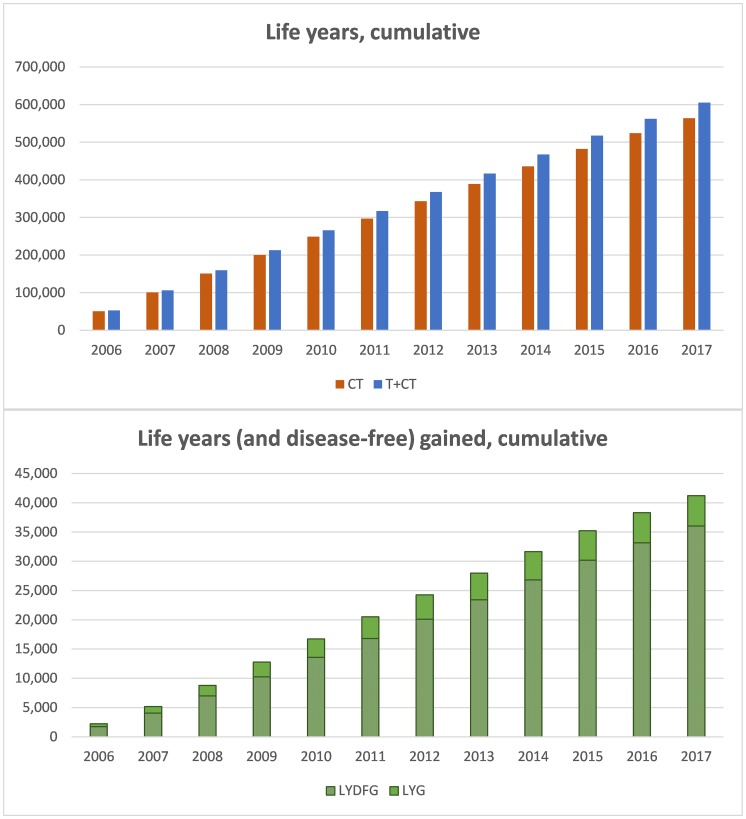
Cumulative results of life years and disease-free life years, follow-up until 2035. (**A**) cumulative life years in the CT and T+CT scenarios, cohorts 2006–2017 (follow-up 2035). (**B**) Cumulative life years gained (disease-free life years shown as dotted bars), cohorts 2006–2017 (follow-up 2035). Abbreviations: CT: chemotherapy; T+CT: trastuzumab plus chemotherapy; LYG: life years gained; DFLYG: disease-free life years gained.

Figure 1 shows the cumulative life years and disease-free life years which the cohorts from 2006 to 2017 would afford up until 2035 in both scenarios, as well as the difference between them, which represents the added clinical benefit of trastuzumab in real life clinical practice in Spain.

Considering the 41,221 additional life years which trastuzumab has afforded and will continue to afford (follow-up until 2035), and taking into account that 35,851 women have been treated to date (cohort 2006–2017), the life expectancy for a patient with HER2+ eBC will have increased 1.15 years. In the same way, the DFLYG with trastuzumab can be calculated. In this case, trastuzumab would extend the disease-free period by one year for each patient.

### Secondary objective: economical results

The total direct costs associated to the sum of 605,358 years of the T+CT scenario for all the cohorts (35,851 women in total) during the analytical period, considering the real use made of trastuzumab according to the market shares, would reach 1,384.02 million euros up until 2035 (€38,604.88 per patient including all direct costs). Most of these costs (61.5%) correspond to the pharmaceutical expenditure of the first year of treatment (851 million euros, €23,738 per patient).

In the hypothetical scenario where trastuzumab is absent, the total direct costs for all the cohorts (35,851 women) would be 503.59 million euros (€14,046.75 per patient) up until 2035. In this scenario, the pharmaceutical expenditure corresponding to the first year of treatment would only represent 16.1% of the total cost (80.98 million euros, €2,259 per patient) - most of the cost (75%) being concentrated in the management of disease relapses.

Therefore, the gain of 41,221 life years thanks to trastuzumab with CT has increased the direct costs by 880.43 million euros (€24,558.13 per patient) since approval of the drug in 2006, and considering follow-up to 2035. Almost 88% (770.06 million euros) of this additional cost is due to the drug expenses corresponding to the first year of treatment. In contraposition to the above, the addition of trastuzumab to CT has reduced the cost of relapses by 12.82 million euros when comparing the T+CT scenario versus the CT scenario. Thus, the overcost of the first year (treatment with trastuzumab) is compensated over time thanks to the savings associated to the decrease in relapses and to the clinical benefits generated in terms of overall survival (OS) and disease-free survival (DFS).

### Secondary objective: cost-effect balance

In this case, in order to strictly compare the incorporation of trastuzumab to standard CT, we do not consider the historical market share of trastuzumab in the T+CT scenario, i.e., in the T+CT scenario all the patients receive trastuzumab as adjuvant treatment instead of applying the real use percentage. If this were not so, the resulting incremental cost-effectiveness ratio (ICER) would not completely determine the incremental effects of adding trastuzumab, since a percentage of patients in the T+CT scenario would not have received the drug.

Table 2 shows the results of the cost-effectiveness analysis with ICERs of €20,644 /LYG and €23,960 /DFLYG.

**Table 2:** Cost-effect balance results, assuming that all patients receive trastuzumab in the T+CT arm, follow-up until 2035

	T+CT	only CT	T+CT vs CT
**Costs**	€ 1,581,553,536	€ 503,587,698	€ 1,077,965,837
**LY**	616,354	564,137	52,217
**DFLY**	534,907	489,916	44,991
**ICER (direct € /LY gained)**			**€ 20,644**
**ICER (direct € /DFLY gained)**			**€ 23,960**

Abbreviations: CT: chemotherapy; T+CT: trastuzumab plus chemotherapy; LY: life years; DFLY: disease-free life years; ICER: incremental cost-effectiveness ratio.

### Results of the sensitivity analysis

The time horizon established in the base case ensures an average follow-up of 23.5 years. As sensitivity analysis, follow-up has been truncated in the year 2030, when the average follow-up is 19 years.

The results of the rest of the scenarios alternative to the base case considered within the sensitivity analyses, along with the results of the univariate analysis, are reported in the Table 3.

**Table 3:** Results of the univariate sensitivity analysis

SA parameters	SA variation	LYG with Trastuzumab	DFLYG with Trastuzumab
Base case		41,221	36,048
Time horizon	2030	30,631	29,761
Efficacy data	HERA	31,533	35,660
Parametric curves from the start (year 1)		40,086	33,383
Temporal preference rate	0%	52,648	43,675
Temporal preference rate	5%	35,662	32,278

Abbreviations: SA: sensitivity analysis; LYG: life years gained; DFLYG: disease-free life years gained.

Since the clinical benefits of trastuzumab in terms of LYG and DFLYG are obtained over time as DFS and OS increase versus CT alone, truncating the results in 2030 shows the greater impact upon the result of the base case, since the follow-up period needed to obtain the clinical benefits is shortened. The rest of the contemplated scenarios with an impact upon the clinical outputs of the model (LYG and DFLYG) have a more moderate influence.

With regard to the sensitivity analysis related to cost variables, quantifying influence upon the incremental cost associated to the T+CT scenario versus CT alone, those related to the estimation of recurrences or their cost exhibit an impact upon the economical outcomes of the base case (an additional 880.43 million euros) of between −0.7% (upper range of recurrence cost) and +12% (possibility of recurrences beyond 7 years). Varying the cost of CT has no effect upon the economical outcomes of the analysis, since it is administered equally in both scenarios. The variation of the temporal preference rate between 0% and 5% has less impact upon the economical outcomes (+1.7 and −0.9%, respectively) than on the results in terms of LYG and DFLYG.

## DISCUSSION

The results of the present analysis, estimating over 41,000 LYG thanks to the addition of trastuzumab to CT in HER2+ early breast cancer women in Spain, shows that the drug substantially improves the prognosis of patients with HER2+ eBC. The main contribution of this study is that it is the first to calculate both clinical and economical outcomes based on the combination of trastuzumab in adjuvant therapy for HER2+ eBC in the real life clinical practice setting in Spain, and on an added basis at population level.

Studies determining the life years that a given treatment has provided to society, are not very numerous. A similar model was developed in the United States to estimate the contribution of rituximab to the treatment of diffuse large B-cell lymphoma (DLBCL) [15]. Danese *et al*. estimated that rituximab in the period between 1999 and 2013 contributed 200,278 LYG in the United States, with an incremental cost of 6.65 thousand million USD during that period.

Although the primary objective of studies of this type is not to determine an incremental cost-effectiveness ratio (ICER) but to quantify the life years which a given treatment has provided to society in the real life clinical practice setting, a ICER may be estimated on the basis of the costs and reported additional clinical benefits afforded. These studies therefore may have some similarity to a conventional cost-effectiveness analysis. In any case, certain relevant aspects of these models differ considerably from the cost-effectiveness models. For example, the latter typically use Markov models with health states involving a hypothetical and homogeneous cohort of patients over a certain time horizon. In contrast, our model involves 12 “real” cohorts (2006–2017) based on Spanish incidence data, and these cohorts are followed-up on to a certain year. In other words, not all the patient cohorts have the same follow-up time. For this reason, the time horizon of the analysis was projected to the year 2035, thereby ensuring a mean follow-up of over 20 years, and being able to correctly record the clinical benefits. In this type of cancer, it otherwise would not have been possible to register the clinical benefits in the more recent cohorts if the analysis had been truncated in the present year.

In the specific case of HER2+ eBC, another important difference between our study and other published cost-effectiveness studies on trastuzumab is the fact that our model is the first to incorporate effectiveness data with a longer follow-up. The trastuzumab cost-effectiveness studies identified in the literature extrapolate OS and DFS from the one- and two-year results of the BCIRG006 and HERA trials. This makes it necessary to view the comparison between our results and those of the previously published cost-effectiveness analyses with caution. Nevertheless, taking into account these differences and the methodological variability among the different studies, the cost-effectiveness ratios calculated in our article are consistent with the findings of most of the cost-effectiveness analyses on trastuzumab in early BC recorded in the systematic review published by Chan *et al*. (2009) [16].

In view of the above, the main contribution of this study is that it quantifies the added clinical value afforded by trastuzumab in the treatment of HER2+ eBC in the real life clinical practice setting in Spain. A recent study has concluded that the use of trastuzumab in HER2+ eBC, together with appropriate screening, is able to modify the epidemiology of the disease, with a significant decrease in the number of patients with metastatic BC [17]. It therefore seems undeniable that trastuzumab has involved a change in the prognosis and treatment paradigm of BC - this affirmation being reinforced by the results of our study at a national population level. Nevertheless, on comparing the results with the general population corresponding to the same age range, it is seen that despite the great advance represented by the introduction of trastuzumab, a total of 98,973 years will still be lost due to HER2+ BC with follow-up of the cohorts to the year 2035. There consequently is still room for new therapies capable of further improving the health outcomes and the prognosis of patients with HER2+ early BC, bringing us closer to the cure of these patients.

In any case, the analysis is not without limitations. Some of them are inherent to pharmacoeconomic models of this kind, which are characterised by a degree of structural rigidity that can make it difficult to offer an appropriate representation of the clinical reality. For example, in order to establish the effectiveness of the treatments, the controlled clinical trials on trastuzumab have been used instead of *real-world data* studies, since the BCIRG006 (base case) and HERA (sensitivity analysis) studies have a longer follow-up, outside the protocol. The results of 25 years of experience with the treatment of early BC in Spain (patients enrolled between 1982 and 2015) have recently been reported, offering broad follow-up on the use of trastuzumab in real life clinical practice. The results obtained indicate an effectiveness of trastuzumab slightly greater than that observed in the open-label phases of the BCIRG006 and HERA trials [18]. Another study has examined the impact of trastuzumab in 284 patients belonging to two cohorts (1996–2005 and 2006–2016), with results that are consistent with the above [19]. Nevertheless, in the developed model it proved necessary to extrapolate the data on DFS and OS. In the case of early BC, although data up to 11 years are available, survival is very high. This complicates parametrisation of the tails of the DFS and OS curves, since many patients have not presented the event in question (disease progression or death).

Another limitation is the fact that trastuzumab is currently used as adjuvant treatment in practically all patients with HER2+ eBC, which makes it difficult to estimate the theoretical effectiveness of the “CT alone” treatment arm. Thus, in order to model the theoretical scenario without trastuzumab, use has been made of the control arms of the pivotal clinical trials, and it has been assumed that efficacy of the latter is maintained over the time horizon of the analysis. Furthermore, in the pivotal clinical trials of trastuzumab, there is crossover between the arms in the open-label phase, and this may cause underestimation of the efficacy of trastuzumab reported in those studies.

Another limitation is a consequence of the estimation of the annual relapses, where certain assumptions have had to be made. For example, based on the opinion of the experts, it has been assumed that there are no further recurrences beyond 7 years after the diagnosis. In addition, these can be calculated using different techniques [20], and we chose the option that best represents clinical practice according to the experts.

The analysis also presents limitations regarding the costs. For example, in the mentioned recurrences it is difficult to establish a global cost comprising all the subsequent treatment lines, since patient management varies considerably among countries and regions, and also over time. Therefore, in our opinion, assuming the cost of the recurrences (equal for all the cohorts along the time horizon, and applied as *one-off cost* at the time of disease progression) to lie within the range obtained by Albanell *et al*. (2016) [21] using two alternative sources for the percentage of patients treated in each line, seemed to be an adequate approach.

Another assumption was that the unit costs of CT remain constant and equal to the present costs (2017). As a result, the costs of CT can be expected to be overestimated in the first years of the analysis. Even so, since CT is administered in both scenarios, the impact of these results would be very low. We likewise did not contemplate the indirect costs associated to productivity losses among the women. These costs were included in the model, though an exploratory analysis showed their impact upon the economical outcomes to be very limited, taking into account the low active population percentage in the women corresponding to the age interval of the analysis. Therefore, despite slight lowering of the incremental cost associated to trastuzumab, we decided not to include these costs in the article.

In order to minimise these limitations, we performed the sensitivity tests that confirmed the robustness of the results obtained. Furthermore, all the assumptions made, the parameters considered, and the results obtained were subjected to validation by the group of experts.

In conclusion, our estimations show that adjuvant trastuzumab has substantially improved the survival of patients with HER2+ eBC, contributing over 41,000 LYG to the Spanish society (over 36,000 DFLYG) in a cost-effective manner. However, the sum of LYG with trastuzumab is still far from the LY estimated for the general population, supporting the need of further advances in HER2+ eBC therapy.

## METHODS

An epidemiological model was developed (using MS Excel 2010) of the benefits and costs associated to trastuzumab as adjuvant therapy in HER2+ eBC since approval of the drug for this indication in Spain in the year 2006.

In annual cycles, the model was based upon the population incidence of early BC to obtain the selected number of newly diagnosed patients forming the model cohorts from 2006 to 2017 (target population), and estimate the incremental survival and incremental costs associated to the use of trastuzumab plus chemotherapy (T+CT), considering its percentage of real use, compared with a hypothetical scenario involving only chemotherapy (CT) in the absence of trastuzumab.

The time horizon of the analysis extended to the year 2035, thus ensuring a mean follow-up of these patients treated between 2006–2017 of 23.5 years, in line with other cost-effectiveness studies in early BC, and allowing us to estimate the total contribution of the treatment [16].

An annual temporal preference rate of 3% was applied for both the costs and effects in future, i.e., referred to projection from the present time (2017) to 2035.

A group of experts in BC (authors of the present article) contributed to design the model, validate the parameters and assumptions made, as well as the clinical feasibility of the results of the analysis.

### Epidemiological data, target population

A Poisson regression model was developed to obtain the BC incidence rates in Spain specific of age, gender and year since 2006 to the present time, based on the Spanish BC incidence data available since 1996–2007 obtained from GLOBOCAN [13]. Applying these rates to the Spanish population figures according to age (25–90 years), gender and year published by the Spanish National Statistics Institute (INE), we calculated the number of new cases of BC diagnosed for the years of the analysis (2006–2017) [22].

After estimating the incident women with BC between 2006 and 2017, we selected those with early disease and amenable to having received trastuzumab, and which were thus regarded as the target population of the analysis. In this respect, we discarded those patients with *in situ* ductal carcinoma (18% [expert opinion]) and those with metastasis at the time of diagnosis (7% [23]).

The prevalence of the HER2+ phenotype in these women was quantified based on the data of the Spanish Society of Pathology (SEAP) from 2013 to date. The percentage of HER2+ in these last years ranged from 16 to 18%, assuming for the years before 2013 the figure reported that year (16.7%). According to the opinion of the experts, it was assumed that all the patients underwent testing.

### Effectiveness

The clinical benefit of trastuzumab in combination with CT versus CT alone in the adjuvant therapy setting of HER2+ eBC was established on the basis of the long-term outcomes of the randomized clinical trials on HER2+ eBC - the experts having considered that these studies with prolonged follow-up periods are representative of real life clinical practice in terms of the benefits associated to the treatment. In this regard, the BCIRG 006 study, the most recent data of which document 10 years of follow-up [5], was used as the base case for parametrising the overall survival (OS) and disease-free survival (DFS) curves used in the model. Therefore, the arm receiving anthracyclines followed by trastuzumab in combination with CT (AC→TH) in the BCIRG 006 study represented the T+CT scenario of the analysis, while the control arm of the BCIRG 006 study (AC→T) represented the hypothetical CT scenario of the analysis. The docetaxel, carboplatin and trastuzumab (TCH) arm of the BCIRG 006 study was not considered, since its use in routine clinical practice in Spain is more limited than that of the AC→TH regimen.

**Figure 2:**
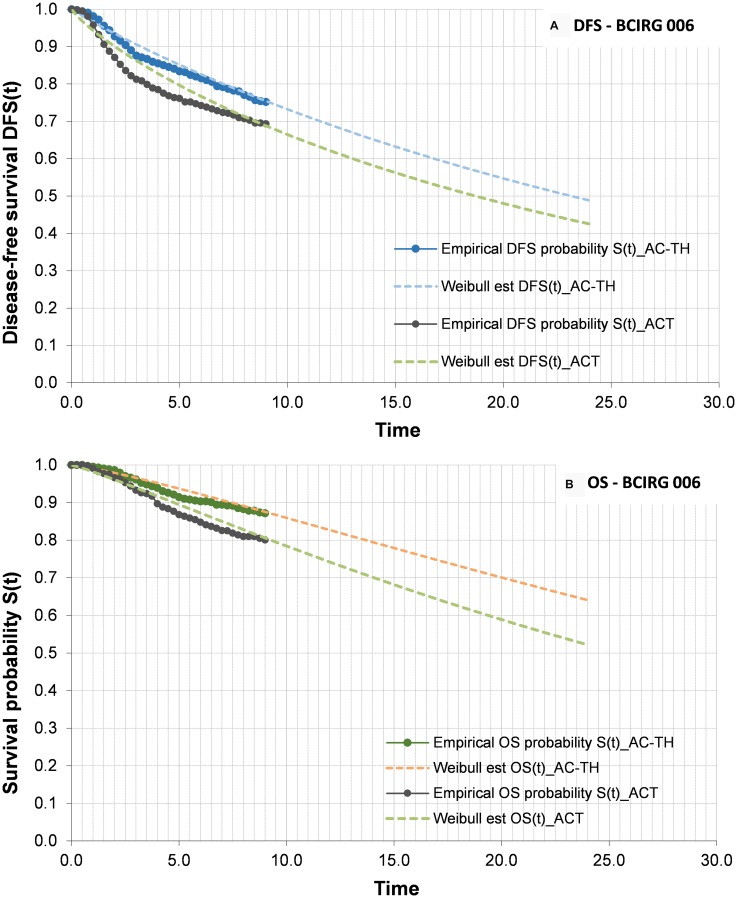
Disease-free survival (**A**) and overall survival (**B**) curves. (A) DFS curves for ACT and AC-TH arms. Bold lines represents KM data, and discontinuous lines the parameterization made to extrapolate the data. (B) OS curves ACT and AC-TH arms. Bold lines represents KM data, and discontinuous lines the parameterization made to extrapolate the data. Abbreviations: DFS: disease-free survival; OS: overall survival; CT: chemotherapy alone; T+CT: trastuzumab plus chemotherapy.

As the time horizon of the analysis was greater than the duration of follow-up in the BCIRG 006 study, it was necessary to adjust the parametric curves to the available efficacy data in order to extrapolate OS and DFS beyond the follow-up period of the mentioned study. Accordingly, parametric models were adjusted with Weibull distribution to the digitalized data of the Kaplan-Meier (KM) curves of the BCIRG 006 study, using the maximum likelihood estimation method [24] for both OS and DFS. Figure 2 graphically displays the OS and DFS curves incorporated to the epidemiological model, differentiating the empirical data (digitalization of the KM curve) from parametrisation (Weibull model).

The OS curves in turn were adjusted according to the Spanish overall mortality by age intervals [22] - only a slight influence in the tail of the curves being observed. Based on these OS curves, the model quantifies the added total life years of the interval 2006–2017 to the time horizon of the analysis (year 2035), for the scenarios T+CT and CT. The difference between the two scenarios provides the life years gained (LYG) associated to trastuzumab. Based on this same reasoning, DFS provides the disease-free life years gained (DFLYG) with trastuzumab.

Furthermore, the model allows us to estimate the number of progressions to the next line of treatment occurring each year, with an impact upon the analysis only in terms of costs. Two methods allow us to quantify the patients in progression each year, based on the area between the DFS and OS curves (*partitioned survival model [PSM]*) or based on calculation of the difference in patients that die or progress each cycle according to DFS minus the deaths according to OS each cycle (*Markov cohort model*) [20]. For the base case, progressions are quantified using the PSM approach. Based on the opinion of the experts, it has been assumed that recurrences occur in the first 7 years after the initial treatment of early BC.

### Management of the disease

All the patients with early BC considered in the analysis received CT, usually accompanied by surgery. We consulted a number of European observational studies to establish the most commonly employed CT schemes [25–28]. Based on the distributions of the chemotherapies reported in these studies, the panel of experts established that the CT schemes which best represent clinical practice in Spain are TC (carboplatin + taxane) and AC-T (doxorubicin + cyclophosphamide + taxane), with a ratio of 25:75, respectively. Paclitaxel was the taxane most frequently used in AC-T, while docetaxel was the taxane most commonly administered in the combination with carboplatin (with a ratio of 75:25 in both cases). The following CT scheme dosage regimens were used:

- paclitaxel 80 mg/m2 weekly during 12 weeks
- doxorubicin 60 mg/m2 during 4 cycles plus
- cyclophosphamide 600 mg/m2 during 6 cycles

Since the aim of the analysis was to establish the real benefit of trastuzumab for Spanish society in HER2+ eBC, the model incorporated the market shares of trastuzumab since its approval for this indication, referred to both the intravenous (IV) formula and the more recent subcutaneous (SC) formula. Posologies for trastuzumab:

- Trastuzumab IV (trastuzumab 150 mg): 8 mg/kg loading dose, followed by 6 mg/kg every 3 weeks, given for 1 year
- Trastuzumab SC (trastuzumab 600 mg): 600 mg every 3 weeks, given for 1 year

**Figure 3:**
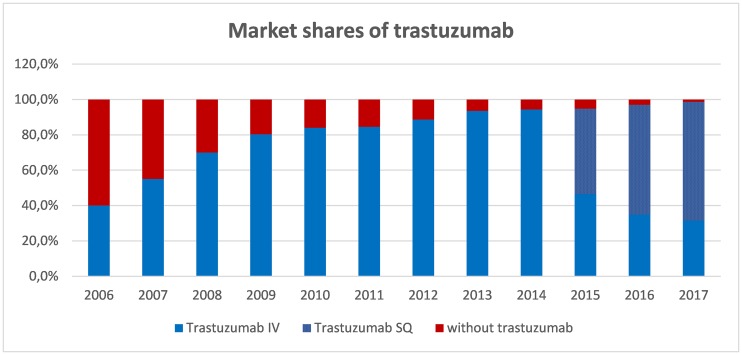
Market shares of trastuzumab as adjuvant therapy in early BC. Blue part of the bar represents market share of trastuzumab. Since 2015, within market share of trastuzumab, spotted part represents SC formulation while smooth part represents IV formulation. Abbreviations: IV: intravenous; SC: subcutaneous.

Figure 3 shows the assumed market share for adjuvant trastuzumab since 2006 (40%) up to present (almost all patients with HER2+ eBC).

### Costs

The analysis considered the direct healthcare costs, classified into the following categories:

- Costs of initial treatment of HER2+ eBC
  ○ Pharmacological (both CT and trastuzumab IV or SC)
  ○ Administration (for IV dosing)
  ○ Monitoring and follow-up
- Costs after progression (recurrences)

The costs of the adverse events associated to the treatments of HER2+ eBC were not included in the analysis, since these costs are already considered within the cost of recurrences. Nevertheless, these costs are considered within the cost of recurrences (see below).

In relation to pharmacological costs we considered a cost per cycle calculated on the basis of the abovementioned CT schemes (with or without trastuzumab) and the hospital dispensation price (notified price to retailer [PTR]) [29]. In the case of drugs where the dose is conditioned to body weight or surface, the calculations were made considering an average weight of 66.4 kg or a body surface of 1.73 m^2^, respectively (corresponding to Spanish women between 45–54 years of age) [22].

On a conservative basis, for the cost of trastuzumab we applied the corresponding deduction set out in Spanish Royal Decree 8/2010 [30], according to the year of the cohort. In other words, no trastuzumab deduction was applied to the cohorts of 2006 to 2010, which predate the publication of RDL 08/2010. Between 2010 and 2014 we applied 7.5%, and from 2014 onwards the current 15% deduction (for IV administration) was applied. The SC formulation is subject to a 7.5% deduction since its marketing in late 2014.

As the variations of the real prices of some of the drugs used in CT from 2006 to date are not precisely known, we considered the current prices for all the cohorts - this implying a conservative position, since the costs of CT in the initial cohorts are probably underestimated.

The intravenous treatments moreover have an associated administration cost of €211.64 corresponding to the visits to the day hospital, obtained from the eSalud healthcare costs database [31]. This cost has been adjusted for each cohort of the model according to the inter-annual variations in the consumer price index (CPI) obtained from the INE [32].

As monitoring and follow-up costs we only included the cost associated to the performance of 3-4 echocardiograms during the year of treatment with trastuzumab and 1 echocardiogram yearly thereafter, the unit cost being €133 [31], likewise adjusted according to the variation in CPI for each year. The panel of experts considered that, apart from the echocardiogram assessment, there is no differential follow-up of the patients according to whether CT combined with trastuzumab is received or not.

Supplementary Table 1 shows the costs of the initial treatment of HER2+ eBC applied during the first year of each cohort, for each of the two analytical scenarios.

The cost of the recurrences was entered in the model as *one-off cost*, i.e., the total cost considered for recurrent disease is fully imputed to the year in which disease progression occurs. In order to estimate total cost after disease progression, we used the cost analysis made by Albanell *et al*., 2016 [21], which estimated the cost of the loco-regional and metastatic relapses (up to a fifth line of treatment). The estimates considered the direct pharmacological and non-pharmacological costs (visits, test, hospital admissions, etc.) and the adverse events associated to the treatments. Within pharmacological costs, pertuzumab in combination with trastuzumab was incorporated from 2014 until 2017, and cost of TDM1 from 2015 until 2017. Based on the cost results of this study, and considering the percentages of patients that receive each treatment line in the analysis of Albanell *et al*., 2016 [21] and also the observational study published by Colomer *et al*., 2016 [33], we obtained weighted costs between €157,658 and €208,682 associated to the recurrences. Therefore, for the base case we used the average of both figures (183,170 €) as total relapse cost, employing these references as ranges of the sensitivity analysis.

### Sensitivity analysis

In order to evaluate the uncertainty of some of the variables used in the analysis and to determine the robustness of the model and the results obtained, we performed a number of deterministic sensitivity analyses, contemplating both methodological alternatives to the base case and modifying certain parameters of the model within a given range.

The following sensitivity analyses were considered:

- Time horizon: results truncated in 2030
- Efficacy data of the HERA study [6]
- Parametric curves from the start (year 1)
- Estimation of recurrences according to the *Markov model approach*
- Possibility of relapses after more than 7 years
- Temporal preference rate for effects and costs(0–5%)
- Unit costs of IV administration and echocardiogram (± 25% over base case value)
- Chemotherapy cost (± 25% over base case value)
- Cost of recurrences (range €157,000 - €209,000)

## SUPPLEMENTARY MATERIALS



## Abbreviations

BC: Breast cancer
HER2: human epidermal growth factor receptor 2
TNM: Tumor–Node–Metastases
LYG: life years gained
DFLYG: disease-free life years gained
OS: overall survival
DFS: disease-free survival
ICER: cost-effectiveness ratio
T+CT: trastuzumab plus chemotherapy
CT: only chemotherapy
