# Supplementary material for: Contribution of trastuzumab to the prognostic improvement of HER2-positive early breast cancer in Spain: an estimation of life years and disease-free life years gained since its approval
Source: Oncotarget. 2019 Jul 2;10(42):4321–32. doi: 10.18632/oncotarget.27039 (PMC6611514; doi:10.18632/oncotarget.27039)
Supplement: Supplementary file 1 [file oncotarget-10-4321-s001.pdf]

Contribution of trastuzumab to the prognostic improvement of HER2-positive early breast cancer in Spain: an estimation of life years and disease-free life years gained since its approval

SUPPLEMENTARY MATERIALS

Supplementary Table 1: Summary of the costs of the initial treatment of HER2+ early BC

Cohort	Pharmacological		Administration		Monitoring		Total	
	CT	T+CT	CT	T+CT	CT	T+CT	CT	T+CT
2006	€ 2,259	€ 30,325	€ 1,060	€ 3,072	-	€ 385	€ 3,319	€ 33,782
2007	€ 2,259	€ 30,325	€ 1,089	€ 3,155	-	€ 395	€ 3,348	€ 33,875
2008	€ 2,259	€ 30,325	€ 1,135	€ 3,287	-	€ 412	€ 3,394	€ 34,024
2009	€ 2,259	€ 30,325	€ 1,151	€ 3,333	-	€ 417	€ 3,410	€ 34,075
2010	€ 2,259	€ 28,220	€ 1,160	€ 3,360	-	€ 421	€ 3,419	€ 32,001
2011	€ 2,259	€ 28,220	€ 1,195	€ 3,461	-	€ 433	€ 3,454	€ 32,114
2012	€ 2,259	€ 28,220	€ 1,223	€ 3,544	-	€ 444	€ 3,482	€ 32,208
2013	€ 2,259	€ 28,220	€ 1,259	€ 3,647	-	€ 457	€ 3,518	€ 32,324
2014	€ 2,259	€ 27,167	€ 1,263	€ 3,658	-	€ 458	€ 3,522	€ 31,283
2015	€ 2,259	€ 26,840	€ 1,250	€ 3,621	-	€ 453	€ 3,509	€ 30,914
2016	€ 2,259	€ 27,025	€ 1,250	€ 2,412	-	€ 453	€ 3,509	€ 29,890
2017	€ 2,259	€ 27,082	€ 1,270	€ 2,137	-	€ 461	€ 3,529	€ 29,680

Abbreviations: CT: chemotherapy alone; T+CT: trastuzumab plus chemotherapy.
